# Population Connectivity and Genetic Assessment of Exploited and Natural Populations of Pearl Oysters within a French Polynesian Atoll Lagoon

**DOI:** 10.3390/genes11040426

**Published:** 2020-04-15

**Authors:** Céline M. O. Reisser, Romain Le Gendre, Cassandre Chupeau, Alain Lo-Yat, Serge Planes, Serge Andréfouët

**Affiliations:** 1 IFREMER, UMR Ecosystèmes Insulaires Océaniens, UPF, ILM, IRD, F-98719 Taravao, Tahiti, Polynésie Française, France; cassandre.chupeau@hotmail.fr (C.C.); alain.lo.yat@ifremer.fr (A.L.-Y.); 2 MARBEC, Univ Montpellier, CNRS, IFREMER, IRD, Montpellier, France; 3 IFREMER, UMR-9220 ENTROPIE, IRD, Université de la Réunion, IFREMER, CNRS, Université de la Nouvelle-Calédonie, Campus IRD, Nouméa BP32078, New Caledonia; romain.le.gendre@ifremer.fr; 4 PSL Research University: EPHE-UPVD-CNRS, USR 3278 CRIOBE, Labex Corail, Université de Perpignan. 52 Avenue Paul Alduy, CEDEX 66860 Perpignan, France; planes@univ-perp.fr; 5 Institut de Recherche pour le Développement, UMR-9220 ENTROPIE, IRD, Université de la Réunion, IFREMER, CNRS, Université de la Nouvelle-Calédonie, Nouméa BPA5, New Caledonia; serge.andrefouet@ird.fr

**Keywords:** *Pinctada margaritifera*, connectivity, population genomics, pearl farming, larval dispersal modelling, MARS3D

## Abstract

In French Polynesia, the production and exportation of black pearls through the aquaculture of the black-lip pearl oyster *Pinctada margaritifera* provide the second largest economic income for the country after tourism. This industry entirely relies on the collection of natural spats from few highly recruiting lagoons. In recent years, pearl oyster producers have experienced variable success rates in spat collection, with significant spatial and temporal variability in spat supply, driving uncertainty in the future of pearl production. This study combines, for the first time in a farmed lagoon, genetic (SNPs), demographic (sex ratio, age), and biophysical data (larval dispersal modelling) to shed new light on population dynamics, connectivity, and spat recruitment in Ahe Atoll, a well-studied pearl farming site. Our results indicate that the geographical structuring of the natural populations and the contribution of both natural and exploited stocks to the production of spats result from the interaction of hydrodynamic features, life history traits and demographic parameters: the northeastern natural populations are older, not well connected to the southwestern natural populations and are not replenished by larvae produced by adjacent exploited populations. Moreover, we observe that the exploited populations did not contribute to larval production during our experiment, despite a sampling period set during the most productive season for spat collection. This is likely the result of a strong male bias in the exploited populations, coupled with a sweepstakes reproductive strategy of the species. Our results warrant further investigations over the future of the northeastern older natural populations and a reflection on the current perliculture techniques.

## 1. Introduction

Aquaculture of marine species usually relies on the production of hatchery individuals or on catches from local natural populations that are then transferred into closed structures at sea for a period of growth. In French Polynesia, the production and exportation of pearls through the aquaculture of the black-lip pearl oyster *Pinctada margaritifera* provides the second largest economic income for the country after tourism. The process of pearl making consists in inserting a piece of mantle tissue from a giver oyster alongside a nucleus into the pearl sac of a receiving oyster. In order to obtain both giver and receiving oysters, the pearl industry entirely relies, so far, on the collection of natural spats (oyster larvae that underwent metamorphosis and started their sessile life stage) on plastic structures called collectors placed in the water column in highly reproductive lagoons. The collected spats are then translocated in pearl farms for subsequent grafting and growth within lagoon waters. Spat production and collection is known to be stochastic in space and time [1,2] but in recent years, pearl oyster producers did report even higher variability in spat collection success in many exploited lagoons [3]. As a result, professionals started to increase the number of collectors placed in lagoons to ensure a minimum collection request [4,5] and questions about the origin of the spats collected and the causes of the variability in collection success became critical for the industry. To address these questions, an understanding of the demographic parameters of exploited and natural populations, as well as of larval dispersal are necessary.

Spat production and collection declines have already been empirically observed in various lagoons (Direction des Ressources Marines. pers. obs.), including in Ahe, a well-studied and historically highly successful collecting lagoon. There, larval dispersal has previously been modeled in an attempt to identify dispersal routes and their correlation with various environmental drivers [6,7,8]. This research allowed for a better prediction of “sink” locations, where larvae would accumulate under particular wind regimes and environmental conditions and contributed to make spat collection more efficient throughout the year. Yet, the overall reduction in the number of spats collected yearly is still observed at present. This decline cannot be attributed to a lack of adult oysters in the lagoon, since the exploited stock in Ahe provides 14 million oysters and the natural stock accounts for approximately 666.000 oysters [4]. Thus, some other processes might be affecting successful reproduction and/or larval survival. 

Recently, concerns have been raised about common pearl farming practices that affect the global sex ratio of the exploited population [4,9]. *P. margaritifera* is a protandrous hermaphrodite, all oysters are male during the first two years of their life and slowly transition to the female sex, from which they will be able to go back and forth (multiple changes from female to male and male to female for a given individual) according to environmental conditions [10]. Globally, a cohort will contain 0% females at one year of age, 27% females at 6 years of age and 41% past 8 years of age [11]. For pearl production, oysters are grafted at around 3 years of age, and most of them will be killed after the pearl harvest (at 4.5 years of age), while fewer used for second grafts will be killed at 6 years of age. The exploited stock is thus very young and mostly composed of males. This sex ratio bias coupled with the much higher density of the exploited stock could prevent efficient mixing of gametes and successful larvae formation, since oyster oocytes can only be fertilized a few hours after being spawned. As such, only a handful of exploited individuals might be successful in contributing to the next generation (i.e. males in the direct proximity of a female individual). This would imply that larval production would essentially originate from the natural populations. In Ahe, a recent study revealed a possible demographic dichotomy among the natural populations: the extrapolation of the sex of individuals using shell size [11] revealed (1) a northeastern area in which older larger female individuals dominated and (2) a southwestern area which contained a mix of males and females [2]. This apparent geographical regionalization of the sex ratios could thus ultimately lead to the decline in the number spat produced and collected, a decrease in genetic diversity, and decreased adaptative capacities of the natural populations, ultimately endangering them. As such, the characterization of the sex ratio in the exploited and natural stocks and the estimation of the contribution of each stock to the spats produced is necessary to understand the dynamic of spat production in farmed lagoons and the reasons of its increased variability.

Nowadays, multiple techniques can be used to characterize the geographical and demographical origin of individuals: direct tagging of individuals using fluorochromes, dispersal modelling, [7,12,13,14,15,16] or parentage analysis through the use of genetic markers [17,18]. Genetic markers also have the advantages to inform about the putative genetic diversity of the parental populations as well as demographic parameters such as the estimated number of adults reproducing in a population. Restriction site-Associated DNA sequencing (or RAD-sequencing) of *P. margaritifera* recently confirmed the level of details that can be obtained with genome-wide markers [19].

Our goal is to shed new light on population dynamics and spat recruitment in Ahe by combining, for the first time in pearl farmed lagoons, genetic, demographic, and biophysical data to attempt to (i) provide baseline and verify estimates of the demographic parameters of the natural and exploited populations of Ahe lagoon, (ii) assess the genetic diversity and heterogeneity of the natural and exploited stock for each sample collected, (iii) assess the potential contribution of each stock to spat recruitment through larval production and dispersion modelling and finally (iv) confront simulations on dispersal and recruitment from the numerical dispersal model with direct observation of spat collection and parentage analysis through genetic markers. 

## 2. Materials and Methods 

### 2.1. Sampling Strategy

**Figure 1:**
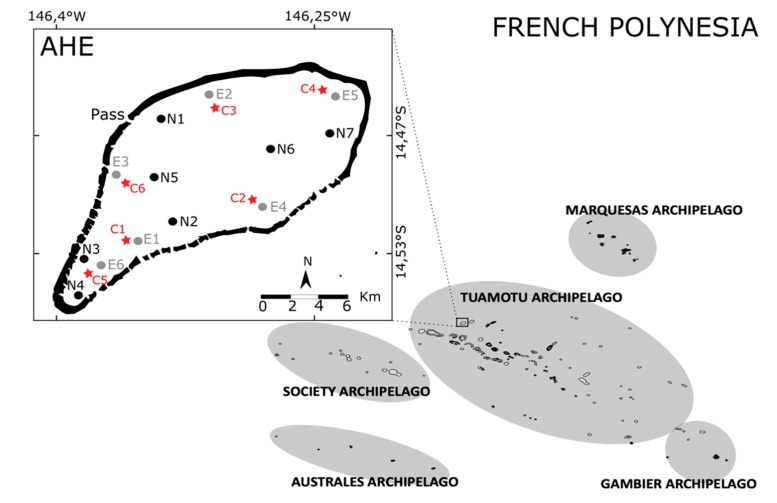
Sampling sites of the study in Ahe Atoll, Tuamotu Archipelago, French Polynesia, showing the location of the six exploited sites (E, in grey), seven natural sites (N, in black) and six collector sites for spats (C, in red).

The study took place in the Ahe atoll lagoon in the Tuamotu Archipelago (Figure 1). The pearl farms and exploited stocks used in this study were chosen in order to maximize the geographic distribution of exploited individuals in the lagoon. For the natural populations, sites were chosen for their estimated population density [4] as to attempt to sample a minimum of 20 individuals per site. Natural (n = 154) and exploited (n = 164) adult *P. margaritifera* sized between 6 and 20 cm dorso-ventral were collected in February 2017 all around the Ahe lagoon, distributed in 7 and 6 geographical sites respectively (Figure 1, Table 1). Only healthy oysters (without visible damages on the shells or mucus accumulation) were sampled.

Spat collectors (18 in total) were placed in the lagoon in the second week of February 2017 in three geographic locations commonly used for collection by professionals (South West lagoon) and in three additional locations to cover the whole study area. Assuming a mean pelagic larval duration of 25 days [6,20,21], these collectors had the opportunity to collect any larvae spawned between approximately three weeks before their positioning and three weeks before their removal. Collectors were sampled at the end of May 2017 and spats of at least 3 mm were kept (to allow proper identification of the species). In total, 41 spats were collected throughout the lagoon.

Shell size was measured using a flat iron ruler and a caliper and sex was also recorded by non-lethal gonadal biopsy and visual observation of the gametes. Exploited individuals were directly bought from the professionals in pearl farms. Since *P. margaritifera* wild populations are protected in French Polynesia, we obtained the permission from the government (Direction des Ressources Marines. DRM) to perform non-destructive sampling of the populations. Sampling was done by SCUBA: the individuals were first extracted from their substrate and brought to the surface. They were anesthetized on the boat with benzocaine (ChemLab) in a seawater bath of 20 L (final benzocaine concentration: 1.2g.L^−1^). After 15 min of incubation, individuals were sampled by cutting piece of 0.5cm^3^ of mantle tissue and oysters were then placed in a second bath containing lagoon water until awakening (closure of the valve under manual pressure indicating the dissipation of anesthesia). Finally, oysters were repositioned on the substrate they were taken from. 

All tissue samples were preserved in RNALater (Qiagen) (50 mg.mL^−1^) and placed at room temperature for 24 h to allow proper infiltration of the solution in the tissue. They were then placed at −20 °C while in the field and at −80 °C once in the lab, before extraction. For spats, we used the entire individual that we placed in individual 1.5 mL Eppendorf tubes and delicately cracked the shell using a clean pestle before adding 200 µL of RNALater (Sigma Aldrich).

### 2.2. DNA Extraction and Library Preparation

DNA was extracted with a commercial Qiagen kit (Blood and Tissue Kit. 250 samples) following the manufacturer protocol. DNA quality and integrity were assessed with Nanodrop 2000 and gel electrophoresis. A total of 262 individuals met the DNA quality requirements for next generation sequencing (136 exploited oysters. 84 natural oysters. 41 collected spats) and were used for Genotyping by sequencing (GBS) and 500 ng of their DNA was sent to the Plateforme d’analyses génomiques at Université Laval sequencing services (Québec City. Canada) for library preparation. DNA was digested using restriction enzymes MspI and PstI. Individuals were then randomly divided into three libraries of 88 and sequenced on 12 chips (four chips per library) on Ion Proton. 

### 2.3. Read Processing and SNP Calling

All bioinformatic analyses were performed on the DATARMOR cluster of Ifremer. GC content, duplicated and overrepresented sequences and quality score of raw reads obtained were evaluated using FastQC. Trimmomatic [22] was used to filter out short (less than 100 pb) and low quality reads (mean phred score lower than 10 in a 4 pb window). Remaining reads were subsequently trimmed at 100 bp and demultiplexed using the process_radtags module of STACKS v2 [23]. Reads were mapped on *P. margaritifera* genome using BWA program with default options [24]. Aligned reads were then filtered using SAMtools [25] and unmapped reads (reads with multiple/chimeric alignments) as well as with a poor quality mapping (MAPQ < 5) were removed. We used Freebayes [26] to perform genotype calling with the following parameters: use-best-n-alleles set at 4 and min-mapping-quality set at 30. We also asked for the genotype qualities to be reported. The VCF file generated by Freebayes was filtered using VCFtools [27], BCFtools [25] and VCFlib in order to 1/ keep only the genotypes with a minimum coverage of 10; 2/ keep only the SNP category and only bi-allelic SNPs; 3/ discard loci with a minor allele frequency less than 0.01 and with more than 10% missing genotypes. We also used a custom script to remove loci with “complex events” (i.e. composite insertion and substitution events). Finally, individuals that had more than 15% missing genotypes were removed from the analysis: one individual from N6 and 3 individuals from C1 were removed. All scripts used here are available in a Github repository (see Supplementary Material).

The sequencing produced a total of 976 million reads. After adaptor and quality trimming 875.729.388 million reads were retained (89.7%). After demultiplexing, 836.267.245 reads (95.4%) were kept. These reads were mapped on the *P. margaritifera* genome and assembled into 453.322 loci. After filtering based on type of feature, minimum depth of sequencing per locus per sample and minimum allele frequency, 13.408 loci were retained for population genetic analysis containing 4.2% of missing genotypes.

### 2.4. Detecting SNPs under Putative Selection

As inferences of population genetics parameters can be influenced by loci not following a neutral model of selection, we investigated for the presence of putative loci under selection in our dataset. We used BayescanV2.1 [28] with a prior model of 10.000, with 10.000 iterations and 200.000 burn-in steps. This allowed us to build a dataset composed of neutral loci for the remaining population genetics analyses.

### 2.5. Genotype Imputation

Some analyses cannot handle the presence of missing data (4.57% missing genotypes in our dataset). We performed genotypic imputation using the *radiator* R package using the “on-the-fly” random forest method. In order to avoid biases in the estimation of the genotypes we first investigated population pairwise genetic differentiation using the neutral set of loci: we estimated F_ST_ coefficients using the *assigner* R package [29]. Significance of differentiation levels was tested using 5000 bootstraps, and 95% confidence intervals (CI) were obtained. Two populations were deemed significantly differentiated if the lower 95% CI interval did not cross 0.001.

### 2.6. Population Genetic Analysis

All of the following analyses were carried out on the neutral imputed and on the neutral non-imputed datasets in order to observe any discrepancies. We used VCFtools to extract relative heterozygosity levels for each individual (number of heterozygous sites divided by the number of polymorphic sites listed in the VCF) and estimate levels of relatedness using the method of Yang et al. [30]. Specifically, the method calculates the unadjusted Akj statistics which is expected to range between 0 for unrelated individuals and 1 for an individual with itself. The presence of differences in heterozygosity levels among groups was tested using the Kruskal-Wallis test in the R package stats. Dunn post hoc test was then performed with the *dunn.test* and *FSA* R packages to identify outlier groups. Pairwise F_ST_ coefficients were obtained with the *assigner* R package. We calculated inbreeding coefficients of each individual with the *related* R package [31] using the *guellergt* method and obtained 95% confidence intervals with 1000 bootstraps.

To assess the number of putative genetic clusters in the dataset, we first performed an unsupervised clustering using the find cluster function of Adegenet and selected the optimum k groups using the Aikake Index Criterion (value of k minimizing AIC). Then, using the k clusters as grouping variable, we performed a Discriminant Analysis of Principal Components (DAPC) with the *Adegenet* R package [32]: first, we used the *xval* function to investigate the optimum number of principal component axes to retain for the analysis (here 80). We then performed a DAPC according to the grouping defined by the *find.cluster* algorithm using all discriminant axes available (number of groups k minus one), followed by a composition plot (structure-like) showing the assignation of each individual to the k clusters. 

All R codes and datasets used in this study are available in a Github repository (see Supplementary Material).

### 2.7. Larval Dispersal Modelling

*P. margaritifera* is a broadcast spawner that releases gametes in the water column, which upon fertilization will produce a D larva in the first 24 h post fertilization. The pelagic larval duration (PLD) of *P. margaritifera* was estimated around 21 to 25 days in hatchery conditions [20], though it could vary from 15 to 30 days in environmental conditions commonly found in French Polynesia (e.g., temperature, food concentration) [21]. Larvae usually disperse through drifting in the lagoon currents, though they have the capacity to do vertical migration in the water column [3].

Larval dispersal modeling was carried out using a revised version of the Ahe lagoon MARS3D hydrodynamic model previously described in Dumas et al. [33]. Enhancements of Ahe configuration included several parameters (e.g., mesh resolution, boundary and atmospheric parameters, influence of oceanic swell in hoa fluxes, etc.) and calibration/validation simulations were performed and compared to the 2008–2009 dataset [33] to ensure improved realism of simulated hydrodynamic features. Briefly, the geographic extent of the atoll configuration remained roughly the same, but mesh grid size was refined from 100 m down to 77 m on the horizontal plane and on the vertical axis, and the number of sigma layers has been increased to 50 (originally 23). Open boundary forcing (tide, currents, temperature and salinity) were obtained from a nested MARS3D parent configuration covering the Tuamotu Archipelago at ~ 1920 m resolution for the coarser grid and a finer grid at ~380 m centered on Ahe Atoll and it neighbor Manihi Atoll. Realistic atmospheric forcing was prescribed using the ERA5 reanalysis dataset [34] covering the whole Earth with a 30 km grid. Lastly, oceanic swells are now taken into account and fluxes in hoa have been improved and are now computed online using a combination of water levels (oceanic and lagoon) and wave parameters. It is important to re-emphasize that both the natural and exploited populations were under similar abiotic environmental conditions. since the farmed oysters are maintained in the lagoon waters.

The simulations were set up to match the field sampling for the exact same period (2017/01/25 to 2017/05/28 and forced with realistic conditions as mentioned above. From each sampling station for both natural and exploited samples, 50.000 larvae were numerically released within a square of 9 cells per side (approx. 700 m) and between 5 and 10 m from the surface. Fifteen different cohorts have thus been released weekly from the 25/01/2017 to the 03/05/2017. Larval behavior was implemented including nychthemeral variations, the same way as in Thomas et al. [3] and each cohort has been tracked and saved hourly. At each collecting station (C1 to C5) the number of larvae arriving in the vicinity of the collectors were counted hourly, considering a threshold PLD period of 25 days. Larvae were included in the census if they were within 500 m of the collector. The 500 m distance came from numerical simulations and a sensitivity analysis performed by Sangare [35] who compared larval counts from simulations with in situ larval census for Ahe lagoon for a different experiment and period of time (in 2007). The 500 m distance provided the highest correlations. A connectivity matrix between each source, natural (N1-N7) or exploited (E1-E6), and each collector (C1-C5) was computed for each cohort. The cumulated total, after summing all cohorts, provided a time-integrated connectivity matrix for the entire period while still being able to assess each cohort individually if needed considering their period of dispersal could occur during different environmental conditions (in particular wind). 

## 3. Results

### 3.1. Size and Sex of Sampled Individuals

**Figure 2:**
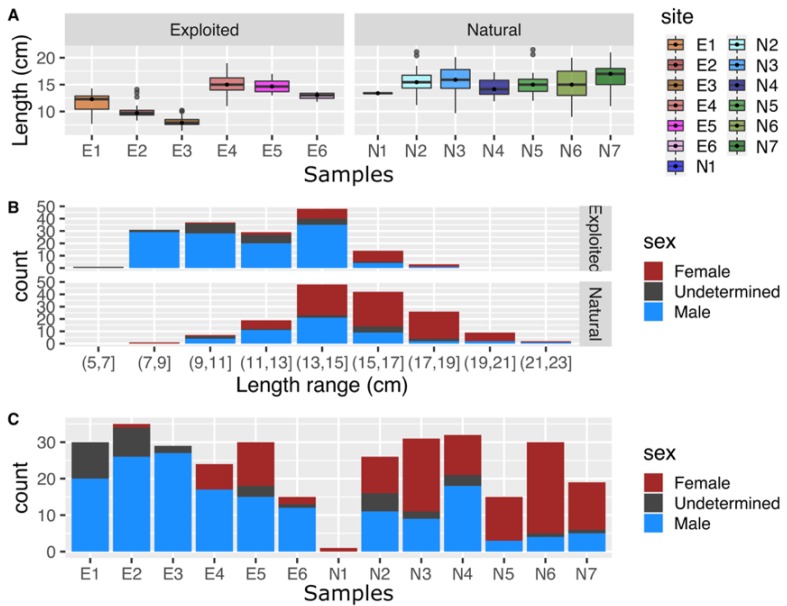
Distribution of (**A**) the size (length in cm), (**B**) the sex ratio of individuals by length class in the exploited and the natural sites, and (**C**) the sex ratio of individuals in all sampled sites.

Despite our best efforts to obtain a homogeneous distribution of individuals’ lengths across the different sampled populations, the size variation across the exploited samples was higher than that of the natural samples (Figure 2A). The natural samples were more homogeneous in the distribution of individual length and was on average longer than those of the exploited samples.

The sex ratio of the natural and exploited samples also differed drastically, with more females being observed in the natural samples (sex ratio between 0 and 0.44 for the exploited samples, versus 0.38 and 1 for the natural samples; Figure 2B. Table 1). When controlling for age differences through the shell length proxy, the natural samples still contained more females for equivalent age/size than the exploited samples (Figure 2C). 

Among the six sets of collectors deployed in the lagoon, the set #6 could not be located in May and was considered lost. From the five collectors left, we collected a total of 41 individuals (21, 8, 7, 4 and 1 individual from C3, C5, C1, C4 and C2 respectively).

### 3.2. Detecting SNPs under Putative Selection and Population Genetics Analysis of Neutral Loci

Bayescan results indicated that 8 loci were putatively under selection (Supplementary Figure S1). These loci were located on 7 different scaffolds: scaffold1138, scaffold1188, scaffold1960, scaffold2247, scaffold3865, scaffold5372 and scaffold6833. These loci had positive α values, indicating a possible divergent selection regime and were removed from subsequent population genetics analyses. 

**Figure 3:**
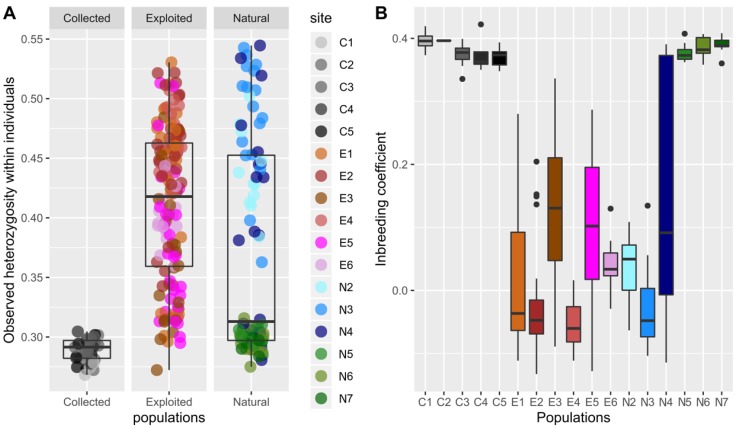
(**A**) Individual relative heterozygosity levels according to the source stock and sites; (**B**) inbreeding coefficients calculated for all individuals.

Both the imputed and non-imputed dataset gave the same results for the following analyses and as such only the results from the imputed dataset will be discussed here. Levels of heterozygosity among the different sites were highly different (Kruskal-Wallis *p*-value < 2 × 10^−16^. Figure 3A). The Dunn post hoc test revealed that these differences occurred among all the three groups (exploited, collected and natural; Supplementary Table S2). To take the analysis further, we divided the natural sites in two groups: the northeastern group (N5, N6 and N7) and the southwestern group (N2, N3 and N4). The test indicated that heterozygosity differences occurred between the exploited and the collected spats group and between the exploited and the natural northeastern group. The collected spats and natural northeast group were deemed similar, as were the exploited and the natural southwestern group ( Supplementary Table S2). 

Inbreeding coefficients show a similar pattern to that of heterozygosity (Figure 3B), with collected spats and the northeastern natural samples being more inbred than the exploited and southwestern natural samples. Pairwise F_ST_ also showed the presence of two groups, one group composed of the northeastern natural and all collected spats samples, and one group composed of the southwestern natural and all exploited samples (Table 2). A few exceptions to this pattern included non-significant F_ST_ values between N2 and N6, and a significant F_ST_ value between E6 and E1, E3, E4 and E5; a significant F_ST_ value between E5 and E3; a significant F_ST_ value between E3 and E1 and E2 (Table 2). The largest F_ST_ values were obtained between the collected spats and the exploited and southwestern natural samples (Table 2). 

**Figure 4:**
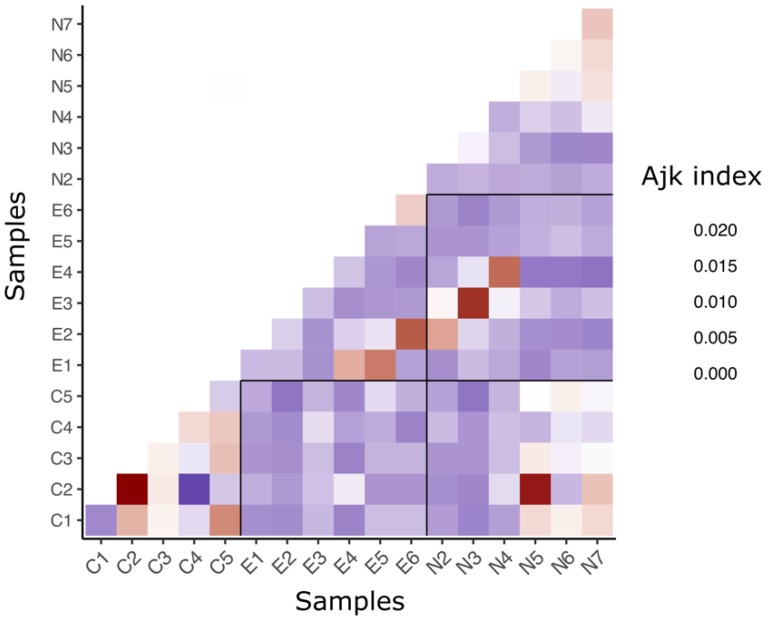
Averaged pairwise relatedness index (Ajk) within and among samples.

Relatedness indices showed that the collected spats were on average more related to each other than the other samples, as were the samples from the northeastern natural populations (Figure 4). Exceptions to this are the C1 and C5 spats, and the E6 samples. C1 spats and C5 spats had a low level of intrasample relatedness but are similar to each other. The E6 samples were more related to each other than the other exploited samples (Figure 4). Also, there was a high variability in the levels of relatedness among samples. And notably for the collected spats.

**Figure 5:**
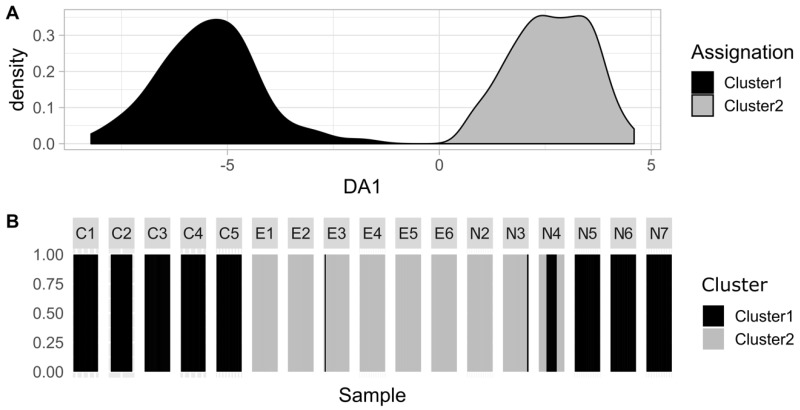
Discriminant Analysis of Principal Components (DAPC) results with grouping definition from the unsupervised clustering: discrimination of the two groups identified by unsupervised clustering (**A**) and assignation of the individuals to the two groups according to their sampling site (**B**).

DAPC cross validation defined an optimum of 170 PC axes to be conserved for analysis. The unsupervised clustering identified 2 main groups in the neutral dataset, constituted of 174 (Cluster 1) and 84 (Cluster 2) individuals (Figure 5A). When overlaying the populational origin of the individuals to the two clusters, individuals were partly separated according to geographical sites and partly according to samples (Figure 5B). Cluster 1 was composed of all the collected spats, all the individuals from northeastern natural samples (N5, N6 and N7), one individual from E3, one individual from N3 and 8 individuals from N4 (Figure 5B). Cluster 2 however contained almost all the exploited individuals as well as the southwestern natural samples N2 and N3, and 12 individuals from N4 (Figure 5B). The collected spats were thus all genetically attached to cluster 1, so to the natural northeastern samples (Figure 5B).

### 3.3. Larval Recuitment Modelling

**Figure 6:**
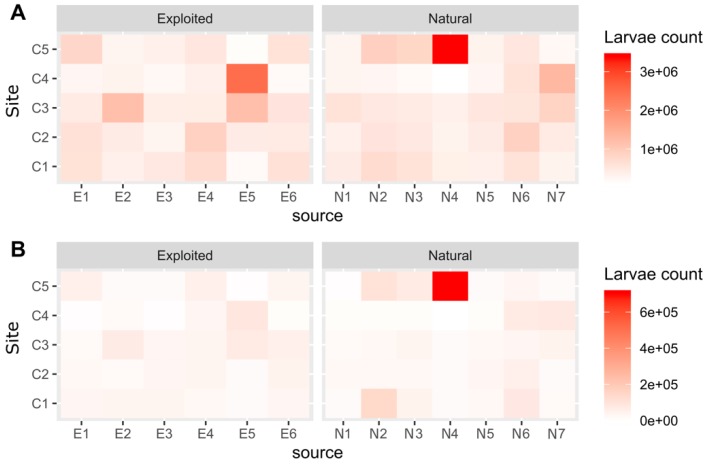
Heatmap showing (**A**) the accumulated larvae (sum of the larvae that settled on a collector for all of the 15 cohorts modelled) that were produced by a given source population (x axis) and that settled onto a given collector (y axis); (**B**) the results for the cohort 12 that best fitted the patterns of dispersion derived from genetic data.

The cumulated (sum of 15 cohorts) connectivity matrices corresponding to the modelled trajectories of larvae generated between the 25th January and the 3rd May 2017 are shown in Figure 5 and Supplementary Figure S2 (for cohort level heatmaps). The pattern of dispersal of larvae varied greatly among the different cohorts. Although two routes seem to be dominant during our experiments (larvae produced by E5 and settling to C4, and larvae produced by N4 and settling to C5), we see that all the collectors potentially recruited individuals from both the natural and the exploited stocks (Figure 6A). When concentrating on the cohorts. the recruitment on C3 was the highest for larvae produced by N4, N5, N6 or N7 for cohort 12. For this same cohort, recruitment could have also occurred for larvae produced by the exploited stock as well. Overall, cohort 12 best fitted the patterns of dispersion derived from genetic data (Figure 6B).

## 4. Discussion

Our study used a combination of life history parameters. Genetic data and modelling to better understand the population dynamics and assess the demographic parameters of the natural and exploited populations of pearl oysters. As well as their interaction and potential respective contribution to larval and spat production in Ahe lagoon.

### 4.1. Demographic and Genetic Analysis of the Exploited Stock

The exploited populations showed heterogeneity in the distribution of individuals’ size across sampled populations. This discrepancy might be due to bias in our sampling material, which was directly bought from the available stock that the professionals used in their farms during our sampling period. However, it could also reflect the two types of aquaculture activities occurring in Ahe. Indeed, the lagoon is exploited by professionals who raise spat collected in the lagoon until the age of grafting (they will have a stock of small sized individuals), and other professionals who use ready-to-graft oysters to produce cultured pearls. Some professionals do both activities while others specialize in one. With both activities occurring in the lagoon, the bimodal distribution of shell sizes of the exploited stock could be expected.

The exploited population showed a strong bias in sex ratio, with most populations being dominated by the male sex. This bias could be further aggravated by the higher number of undetermined individuals in the exploited stock (individuals at mature stage for reproduction but for which no gametes could be observed in the gonad biopsy), especially for individuals of size 9–15 cm in E1, E2 and E3. This absence of gametes in those individuals could be due to (i) spawning occurring a few days prior to the biopsy, but since spawning is mostly triggered by environmental conditions, it should also have affected natural populations, or (ii) an interruption of gametogenesis, because of stress caused by aquaculture practices (suspension on moving culture ropes in the water column, regular epibiont cleaning, etc.) [9] or other unknown disturbances (food depletion, etc.). The stress hypothesis is more likely, as stress was previously reported to affect gametogenesis in pearl oysters [9,10]. 

Genetically, the exploited samples formed a homogeneous group, with all populations resembling each other, which can be expected since pearl farms do buy their oysters from the same professional in Ahe.

### 4.2. Demographic and Genetic Analysis of the Natural Stock

The natural populations had a relatively unimodal distribution of shell sizes and individuals were on average larger than the exploited individuals. This larger size could reflect a bias due to our sampling technique (diving) and the difficulty to observe small individuals in the wild [4], although we were able to find wild individuals between 7 and 10 cm, especially in the southwestern part of the lagoon. The natural samples also showed variability in sex ratio, with a dominance of large female individuals in the northeastern ones and a balanced sex ratio in the southwestern ones. Our results are congruent with previous sex ratio predictions in the natural samples based on shell size distribution [4].

While no genetic differentiation was present among the exploited samples, we found significant genetic structuring and heterozygosity differences within the natural samples, with the presence of two genetic clusters separating four southwestern and three northeastern samples. The four southwestern samples were genetically similar to the exploited samples. The similarity between the southwestern and the exploited stocks and their differentiation to the northeastern samples is not related to the distance between natural and exploited sites, since pearl farms are also distributed in the northeastern part of the lagoon. As such, another process is at play to explain the peculiar genetic signature of the northeastern natural samples. Our results seem to indicate that the northeastern samples are not well connected to the rest of the lagoon: they are made of larger, older individuals, mostly females (up to 86%), all with a specific “northeastern” genetic signature, meaning they are not replenished by larval recruitment from either the exploited stock, or the southwestern natural stock. Previous research using modelling of larval dispersal showed the existence of two hydrodynamic systems in Ahe, separating the northeastern lagoon from the southwestern lagoon [7]. The model showed that wind and resource distribution patterns averaged over the years favored two dominant dispersal dynamics: (i) self-recruitment within each hydrodynamic system and (ii) east to west transfers, but very rarely west to east transfers [7,8]. The southwestern natural populations thus appear to be sink populations for the dispersion of larvae, as exemplified by the southwestern most population (N4), composed of individuals belonging to the two genetic clusters identified in this study. However, while the hydrodynamic barrier could have contributed to the isolation of the northeastern populations, these natural populations are also surrounded by farms with significant exploited stocks that could contribute to larval production and self-recruitment. However, we did not find a single oyster with an exploited genetic signature in the natural northeastern populations, as if actually, the exploited stock was not there, or never contributing to larval production in the northeast. The ability of the exploited stock to efficiently produce larvae is thus challenged by our findings, which warrants detailed analysis of the spat that were collected during our experiment.

### 4.3. Contribution of the Exploited and Natural Populations to Spat Production in Ahe

The genetically similar exploited and natural samples of the shallow southwestern lagoon where both spat collectors and rearing lines are abundant confirm that this is a likely an area promoting population mixing and contributing to homogenization. The shallow lagoon and overall balanced sex-ratio when considering both natural and exploited samples likely favor effective reproduction in this area and genetic homogenization. This does not prevent, however, the import of larvae from other sectors as shown by genetic data in this study and by modelling data [5,6]. However, while a previous simulation of the contribution of the exploited and natural populations to the production/recruitment of spats in Ahe indicated a possible fourfold contribution of the exploited stock to larval production [8], all the spats that were collected during our experiment were genetically assigned to the northeastern natural populations, even those collected on collectors placed in the southwest of the lagoon. The absence of spats produced by the exploited stocks could come from the positioning of our collectors, which might not have been in the path of a larval cloud originating from the exploited populations. Our modelling results did confirm connections between the natural stock sources N6-N7 and the collectors C2-C3-C4, in agreement with genetics data. Conversely, the same cohort suggests connections between sources E2-E5-E6 and collectors C3-C4, but none of these possible connections were confirmed by genetics. This indicates that, should larvae have been produced by E2, E5 or E6, collectors placed in C2, C3 and C4 should have been able to recruit them. This suggests the presence of another process limiting recruitment and/or impeding the production of larvae by the exploited stock.

It is possible that only a handful of individuals reproduced during our experiment, all from the northeastern populations. This phenomenon is called “Sweepstakes Reproductive Success” (SRS). SRS states that the chances of a highly fecund marine animal to contribute offspring in a future pool of reproductively mature adults is a process with a few “winners” and many “losers” [36], which means that only a small subset of the adult population could contribute to each larval cohort. Results for the relatedness index showed high variability in the pairwise individual relatedness both within and among samples. In particular, the high variability in relatedness in the spat samples could indicate the presence of more than one cohort originating from a different subset of adults gametes, since some collected spats of the same collector show low relatedness index (e.g., C1), while these same spats are more related to spats from another collector site (C5). These findings coupled with the high F_IS_ coefficients found in the collected spats indicate that SRS could apply to *P. margaritifera*, as it has been now reported in many marine invertebrates. However, SRS cannot be the only explanation for the lack of spats produced from the exploited populations. Indeed, we know that gametogenesis occurred in the exploited populations, since we successfully biopsied individuals. However, the northeastern natural populations do not contain any individuals with the exploited genetic signature, despite their presence in pearl farms in the area. SRS could be an aggravating factor for successful reproduction if coupled with regionalization of sex ratio, as observed here. The exploited stock is male dominated, while the natural stock is female dominated, which is likely to prevent mixing of gametes and successful larval production. This is likely a key factor to explain the slow but steady decrease in spat collecting reported by farmers in Ahe atoll.

## 5. Conclusions

The results presented here show that the geographical structuring of the exploited and natural populations and their contribution to the production of spats is a complex phenomenon resulting from the interaction of hydrodynamic features, life history traits and demographic parameters. The strong geographical regionalization of the sex-ratios in the various populations likely prevent the efficient mixing of gametes, especially for the male dominated exploited populations. Gamete mixing is likely further prevented by a possible sweepstake reproductive strategy of *P. margaritifera*, for which only a handful of individuals do reproduce at a given time. The fact that spat produced during our experiment were only produced by the natural stock also strongly suggests that serious measures have to be rapidly taken for the management and protection of those populations. In addition, a reflection on the pearl farming methods and on the possibility to increase the number of female oysters in exploited populations is to be considered, such as keeping the oldest oysters on the lines instead of killing them or organizing the repopulation of the northeastern lagoon coral pinnacles with young individuals. Indeed, the reproduction potential of *P. margaritifera* in Ahe is far from optimized and could in time jeopardize the recruitment of spats and the activity around the production of Tahitian black pearls in Ahe. 

Finally, this study also highlights the utility of combining modelling and genetic data for the study of larval dispersal and recruitment. Here, we confirm that larval dispersion modelling can realistically predict spat collecting patterns in Ahe when populations are producing larvae. The addition of genetics data increased the precision of the model, indicating which individual cohort was the one more likely recruited on the collectors and also highlighted an inequal contribution of the natural and exploited stock to spat production during the time of the experiment, by allowing assignation of the collected spats to their stock of origin.

## Figures and Tables

**Table 1:** Sampling characteristics of the study. Stock: stock sampled (E: exploited. N: natural. C: collected spats); Site: name attributed to the sampling site; Lat/Long: latitudinal and longitudinal coordinates; Nb samp: number of individuals sampled for morphometry and sex assessment; Nb seq: number of individuals sequenced and in brackets number of individuals removed from analysis based on quality filtering; H_O_: observed heterozygosity; H_E_: expected heterozygosity; F_IS_: estimated F_IS_ (*: significantly different from 0); Female ratio: the number of females divided by the number of successfully sexed individuals.

Stock	Site	Lat. (S)	Long. (W)	Nb Samp.	Nb Seq.	H_O_	H_E_	F_IS_	Female Ratio
E	E1	−14.526920°	−146.354895	30	25	0.352	0.350	0.014	0.000
	E2	−14.444375°	−146.312045°	35	31	0.372	0.353	−0.028	0.037
	E3	−14.494011°	−146.367102°	29	25	0.299	0.337	0.122 *	0.000
	E4	−14.511260°	−146.276230°	24	11	0.386	0.356	−0.060	0.290
	E5	−14.444012°	−146.234397°	30	28	0.312	0.342	0.103 *	0.440
	E6	−14.537236°	−146.374801°	16	16	0.335	0.341	0.031 *	0.140
S	N1	−14.45873°	−146.34138°	1	0	NA	NA	NA	1.000
	N2	−14.52084°	−146.3386°	26	11	0.340	0.345	0.027 *	0.470
	N3	−14.53645°	−146.38179°	31	17	0.372	0.352	−0.029	0.690
	N4	−14.560160°	−146.387200°	32	20	0.299	0.341	0.132 *	0.380
	N5	−14.499860°	−146.354870°	15	11	0.205	0.324	0.338 *	0.800
	N6	−14.478020°	−146.281850°	30	15(1)	0.200	0.320	0.362 *	0.860
	N7	−14.466320°	−146.246520°	19	10	0.199	0.319	0.344 *	0.720
C	C1	−14.526920°	−146.354895°	7	7(3)	0.198	0.321	0.301*	NA
	C2	−14.511260°	−146.276230°	1	1	0.198	NA	NA	NA
	C3	−14.444375°	−146.312045°	21	21	0.200	0.322	0.349 *	NA
	C4	−14.444012°	−146.234397°	4	4	0.202	0.317	0.282 *	NA
	C5	−14.537236°	−146.374801°	8	8	0.205	0.321	0.317 *	NA

**Table 2:** Pairwise F_ST_ calculations. Lower triangle: F_ST_ values; upper triangle: 95% CI intervals (5000 bootstraps). Bold values: significant F_ST_ (95%CI lower interval ≥ 0.001).

	C1	C2	C3	C4	C5	E1	E2	E3	E4	E5	E6	N2	N3	N4	N5	N6	N7
C1	–	0.0000–0.0066	0.0000–0.0000	0.0000–0.0018	0.0000–0.0000	0.0139–0.0181	0.0171–0.0211	0.0074–0.0119	0.0161–0.0211	0.0103–0.0146	0.0133–0.0182	0.0095–0.0146	0.0154–0.0199	0.0071–0.0117	0.0000–0.0016	0.0000–0.0000	0.0000–0.0021
C2	0.0000	–	0.0000–0.0165	0.0000–0.0207	0.0000–0.0194	0.0590–0.0727	0.0705–0.0838	0.0415–0.0565	0.0708–0.0847	0.0447–0.0589	0.0629–0.0779	0.0616–0.0769	0.0682–0.0820	0.0357–0.0512	0.0000–0.0077	0.0000–0.0124	0.0000–0.0159
C3	0.0000	0.0072	–	0.0000–0.0017	0.0000–0.0009	0.0045–0.0061	0.0061–0.0075	0.0036–0.0053	0.0009–0.0031	0.0035–0.0050	0.0042–0.0063	0.0006–0.0029	0.0046–0.0065	0.0019–0.0036	0.0000–0.0023	0.0000–0.0014	0.0008–0.0034
C4	0.0000	0.0079	0.0000	–	0.0000–0.0011	0.0152–0.0195	0.0176–0.0217	0.0087–0.0133	0.0153–0.0203	0.0109–0.0158	0.0160–0.0210	0.0106–0.0158	0.0174–0.0220	0.0067–0.0115	0.0000–0.0028	0.0000–0.0000	0.0000–0.0014
C5	0.0000	0.0088	0.0000	0.0000	–	0.0085–0.0112	0.0115–0.0140	0.0069–0.0098	0.0072–0.0106	0.0055–0.0081	0.0095–0.0127	0.0053–0.0090	0.0112–0.0142	0.0043–0.0073	0.0000–0.0012	0.0000–0.0000	0.0000–0.0041
E1	**0.0160**	**0.0658**	**0.0053**	**0.0173**	**0.0099**	–	0.0005–0.0019	0.0013–0.0025	0.0000–0.0000	0.0000–0.0000	0.0024–0.0040	0.0000–0.0015	0.0004–0.0017	0.0003–0.0016	0.0075–0.0097	0.0048–0.0067	0.0078–0.0102
E2	**0.0191**	**0.0773**	**0.0068**	**0.0196**	**0.0127**	0.0009	–	0.0011–0.0021	0.0000–0.0003	0.0000–0.0000	0.0000–0.0000	0.0000–0.0000	0.0000–0.0009	0.0012–0.0023	0.0088–0.0109	0.0061–0.0078	0.0096–0.0117
E3	**0.0096**	**0.0489**	**0.0044**	**0.0110**	**0.0083**	**0.0019**	**0.0016**	–	0.0000–0.0009	0.0010–0.0022	0.0014–0.0030	0.0000–0.0000	0.0000–0.0000	0.0000–0.0000	0.0040–0.0063	0.0032–0.0051	0.0054–0.0078
E4	**0.0185**	**0.0778**	**0.0020**	**0.0178**	**0.0089**	0.0000	0.0000	0.0001	–	0.0000–0.0000	0.0019–0.0039	0.0000–0.0000	0.0000–0.0008	0.0000–0.0000	0.0041–0.0070	0.0008–0.0033	0.0053–0.0083
E5	**0.0124**	**0.0517**	**0.0043**	**0.0131**	**0.0068**	0.0000	0.0000	**0.0016**	0.0000	–	0.0015–0.0030	0.0000–0.0003	0.0006–0.0018	0.0000–0.0012	0.0060–0.0082	0.0029–0.0047	0.0062–0.0086
E6	**0.0157**	**0.0705**	**0.0052**	**0.0185**	**0.0110**	**0.0032**	0.0000	**0.0022**	**0.0029**	**0.0022**	–	0.0003–0.0024	0.0029–0.0046	0.0021–0.0038	0.0067–0.0094	0.0038–0.0061	0.0071–0.0098
N2	**0.0120**	**0.0693**	**0.0017**	**0.0132**	**0.0071**	0.0006	0.0000	0.0000	0.0000	0.0000	0.0013	–	0.0000–0.0004	0.0000–0.0004	0.0020–0.0051	0.0000–0.0015	0.0034–0.0065
N3	**0.0177**	**0.0751**	**0.0055**	**0.0197**	**0.0127**	0.0010	0.0004	0.0000	0.0000	0.0012	**0.0038**	0.0000	–	0.0006–0.0021	0.0070–0.0095	0.0047–0.0068	0.0090–0.0117
N4	**0.0093**	**0.0434**	**0.0027**	**0.0091**	**0.0058**	0.0009	**0.0018**	0.0000	0.0000	0.0006	**0.0029**	0.0000	0.0014	–	0.0025–0.0049	0.0017–0.0036	0.0030–0.0055
N5	0.0000	0.0000	0.0010	0.0000	0.0000	**0.0086**	**0.0099**	**0.0051**	**0.0056**	**0.0071**	**0.0080**	**0.0036**	**0.0083**	**0.0037**	–	0.0000–0.0015	0.0000–0.0035
N6	0.0000	0.0030	0.0004	0.0000	0.0000	**0.0058**	**0.0070**	**0.0042**	**0.0021**	**0.0038**	**0.0049**	0.0003	**0.0058**	**0.0026**	0.0000	–	0.0000–0.0002
N7	0.0000	0.0057	0.0021	0.0000	0.0020	**0.0090**	**0.0106**	**0.0066**	**0.0068**	**0.0074**	**0.0085**	**0.0049**	**0.0103**	**0.0042**	0.0017	0.0000	-

## Supplementary Materials

All codes used in this study are available online at https://github.com/cmor2207/PERSIST, The following are available online at https://www.mdpi.com/2073-4425/11/4/426/s1, Figure S1: Bayescan Selection results. Figure S2: Heatmaps of the connectivity matrices obtained for each of the 15 cohorts modelled; Table S1: Annotation of the eight SNPs putatively selected. Table S2: Dun’s post Hoc results.
