# Correction: Reisser, C.M.O. et al. Population Connectivity and Genetic Assessment of Exploited and Natural Populations of Pearl Oysters within a French Polynesian Atoll Lagoon. *Genes* 2020, *11*, 426

**DOI:** 10.3390/genes11111358

**Published:** 2020-11-16

**Authors:** Céline M. O. Reisser, Romain Le Gendre, Cassandre Chupeau, Alain Lo-Yat, Serge Planes, Serge Andréfouët

**Affiliations:** 1 IFREMER, UMR Ecosystèmes Insulaires Océaniens, UPF, ILM, IRD, Taravao F-98719, Tahiti, Polynésie Française, France; cassandre.chupeau@hotmail.fr (C.C.); alain.lo.yat@ifremer.fr (A.L.-Y.); 2 MARBEC, Univ Montpellier, CNRS, IFREMER, IRD, Montpellier, France; 3 IFREMER, UMR-9220 ENTROPIE, IRD, Université de la Réunion, IFREMER, CNRS, Université de la Nouvelle-Calédonie, Campus IRD, Nouméa BP32078, New Caledonia; romain.le.gendre@ifremer.fr; 4 PSL Research University: EPHE-UPVD-CNRS, USR 3278 CRIOBE, Labex Corail, Université de Perpignan, 52 Avenue Paul Alduy, CEDEX 66860 Perpignan, France; planes@univ-perp.fr; 5 Institut de Recherche pour le Développement, UMR-9220 ENTROPIE, IRD, Université de la Réunion, IFREMER, CNRS, Université de la Nouvelle-Calédonie, Nouméa BPA5, New Caledonia; serge.andrefouet@ird.fr

**The authors wish to make the following corrections to this paper** [1]**, regarding an error in Figure 6.**

In the original article, there was a mistake in Figure 6 as published. The aforementioned figure does not display the correct modelled connectivity matrices. The corrected Figure 6 appears below. The authors apologize for any inconvenience caused and state that the scientific conclusions are unaffected. The published version will be updated on the article webpage, with a reference to this correction notice.


