# Supplementary material for: Population Connectivity and Genetic Assessment of Exploited and Natural Populations of Pearl Oysters within a French Polynesian Atoll Lagoon
Source: Genes (Basel). 2020 Apr 15;11(4):426. doi: 10.3390/genes11040426 (PMC7230779; doi:10.3390/genes11040426)
Supplement: Supplementary file 1 [file genes-11-00426-s001.pdf]

Supplementary material

Supplementary Figure S1: Bayescan selection results

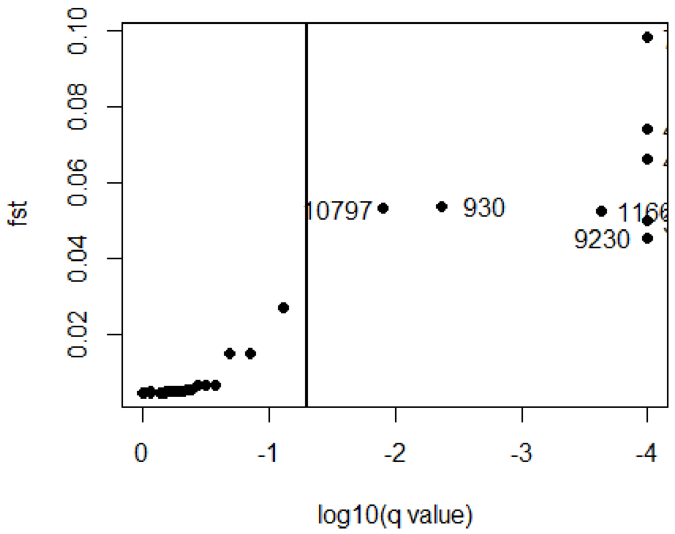

Supplementary Figure S2: heatmaps of the connectivity matrices obtained for each of the 15 cohorts modelled

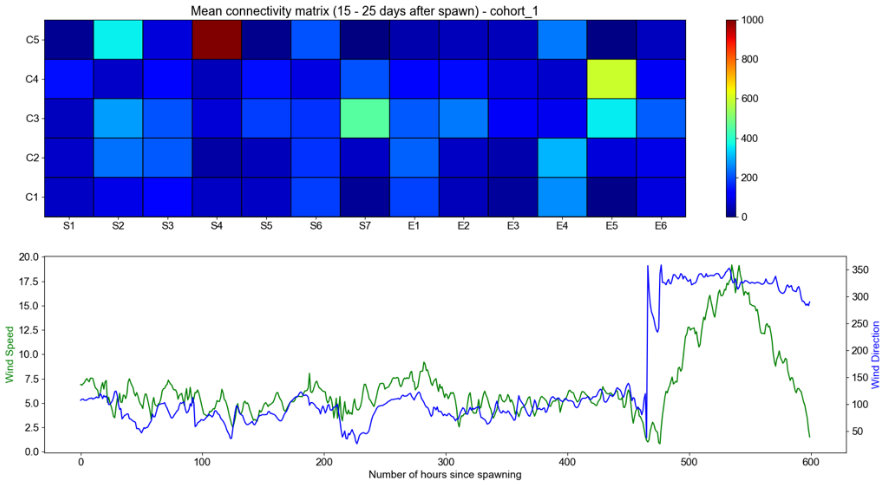

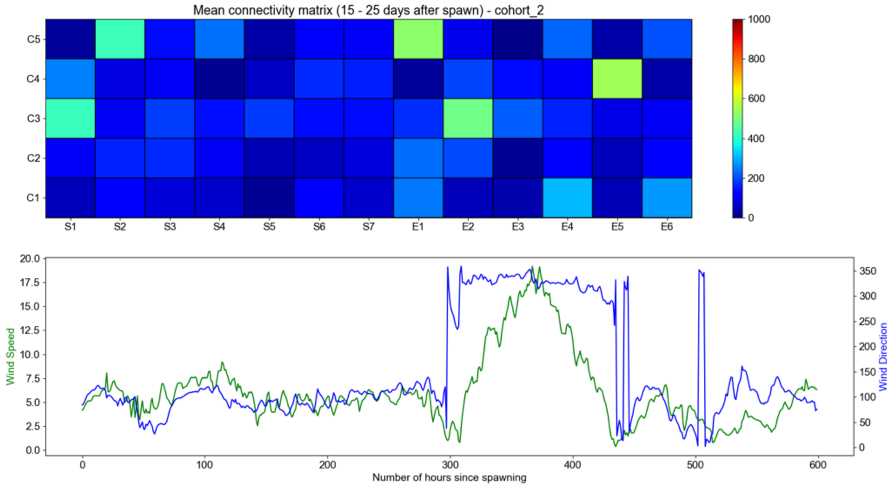
Mean connectivity matrix (15 - 25 days after spawn) - cohort_2
C5
C4
C3
C2
C1
S1 S2 S3 S4 S5 S6 S7 E1 E2 E3 E4 E5 E6
1000
800
600
400
200
0
Wind Speed
Wind Direction
Number of hours since spawning

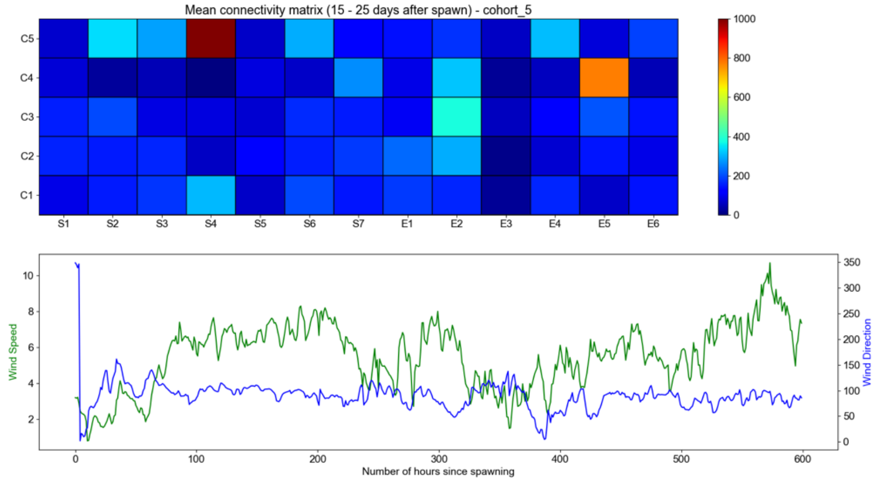
Mean connectivity matrix (15 - 25 days after spawn) - cohort_5
C5
C4
C3
C2
C1
S1 S2 S3 S4 S5 S6 S7 E1 E2 E3 E4 E5 E6
1000
800
600
400
200
0
Wind Speed
Wind Direction
Number of hours since spawning

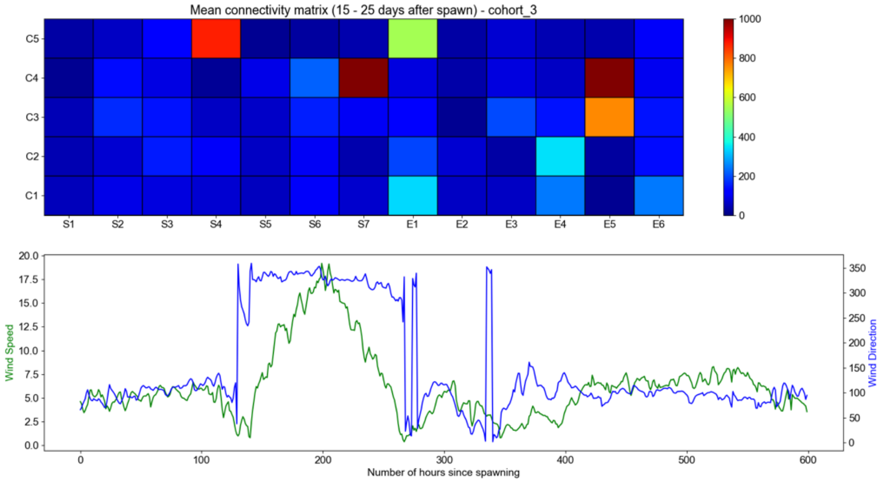
Mean connectivity matrix (15 - 25 days after spawn) - cohort_3
C5
C4
C3
C2
C1
S1 S2 S3 S4 S5 S6 S7 E1 E2 E3 E4 E5 E6
1000
800
600
400
200
0
Wind Speed
Wind Direction
Number of hours since spawning

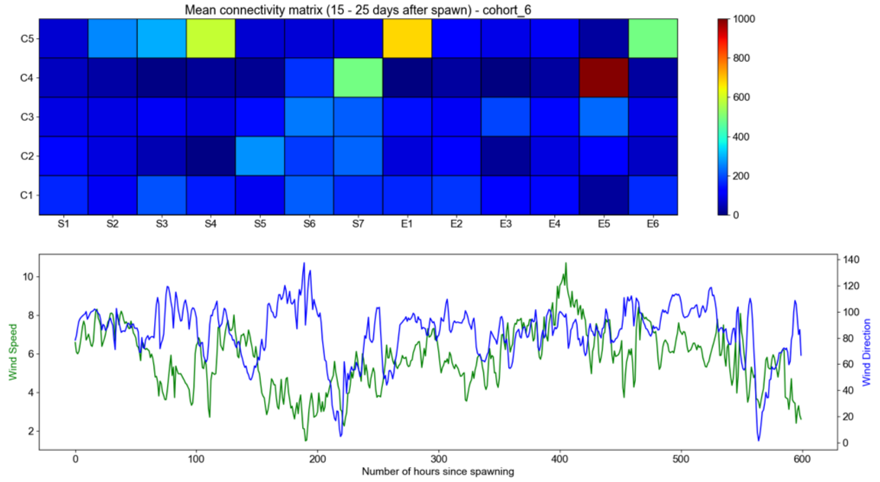
Mean connectivity matrix (15 - 25 days after spawn) - cohort_6
C5
C4
C3
C2
C1
S1 S2 S3 S4 S5 S6 S7 E1 E2 E3 E4 E5 E6
0 200 400 600 800 1000
Wind Speed
Wind Direction
Number of hours since spawning

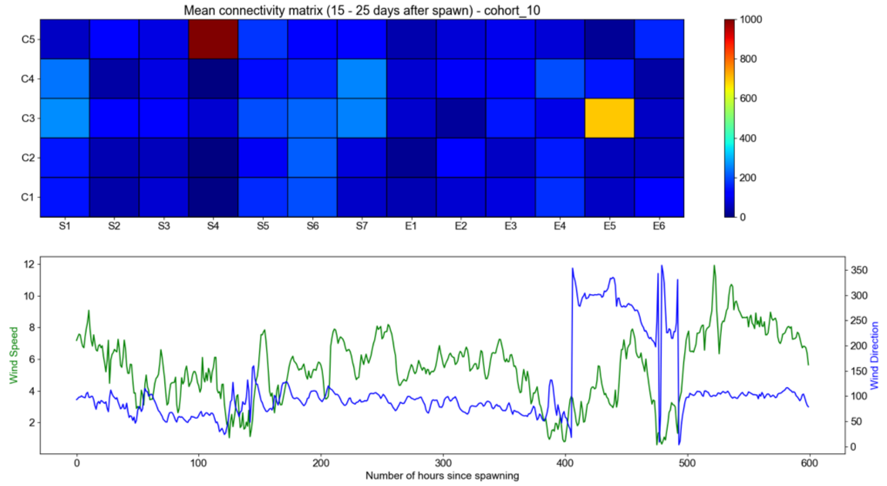
Mean connectivity matrix (15 - 25 days after spawn) - cohort_10
C5
C4
C3
C2
C1
S1 S2 S3 S4 S5 S6 S7 E1 E2 E3 E4 E5 E6
0 200 400 600 800 1000
Wind Speed
Wind Direction
Number of hours since spawning

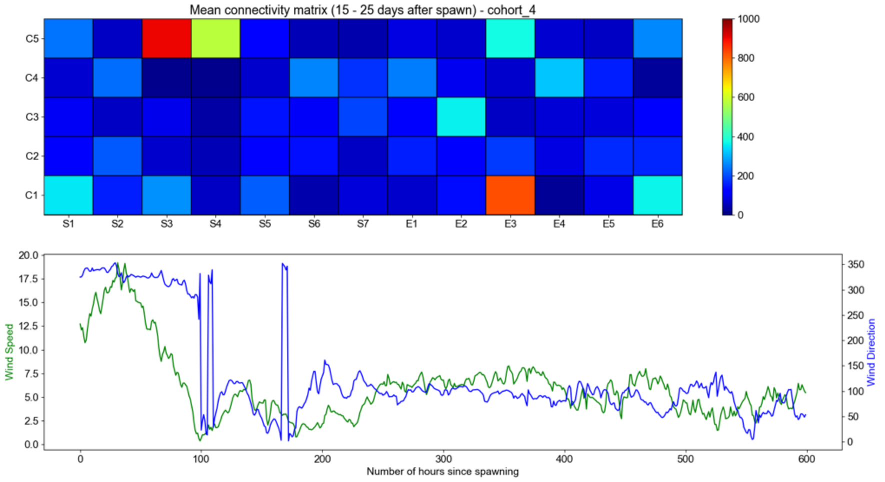
Mean connectivity matrix (15 - 25 days after spawn) - cohort_4
C5
C4
C3
C2
C1
S1 S2 S3 S4 S5 S6 S7 E1 E2 E3 E4 E5 E6
0 200 400 600 800 1000
Wind Speed
Wind Direction
Number of hours since spawning

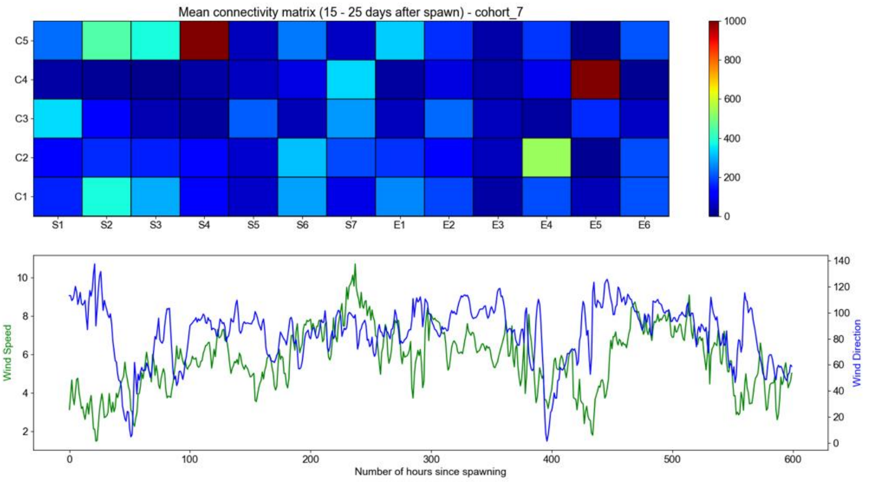
Mean connectivity matrix (15 - 25 days after spawn) - cohort_7
C5
C4
C3
C2
C1
S1 S2 S3 S4 S5 S6 S7 E1 E2 E3 E4 E5 E6
1000 800 600 400 200 0
Wind Speed
Wind Direction
Number of hours since spawning

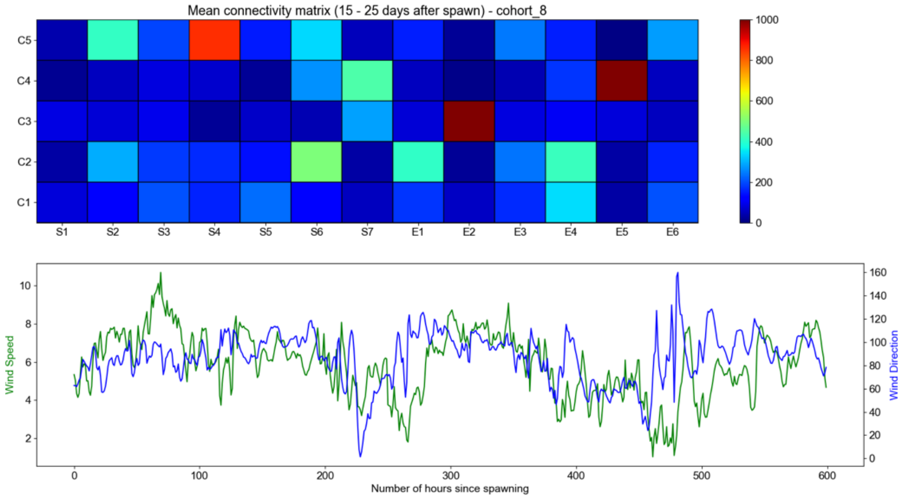
Mean connectivity matrix (15 - 25 days after spawn) - cohort_8
C5
C4
C3
C2
C1
S1 S2 S3 S4 S5 S6 S7 E1 E2 E3 E4 E5 E6
1000 800 600 400 200 0
Wind Speed
Wind Direction
Number of hours since spawning

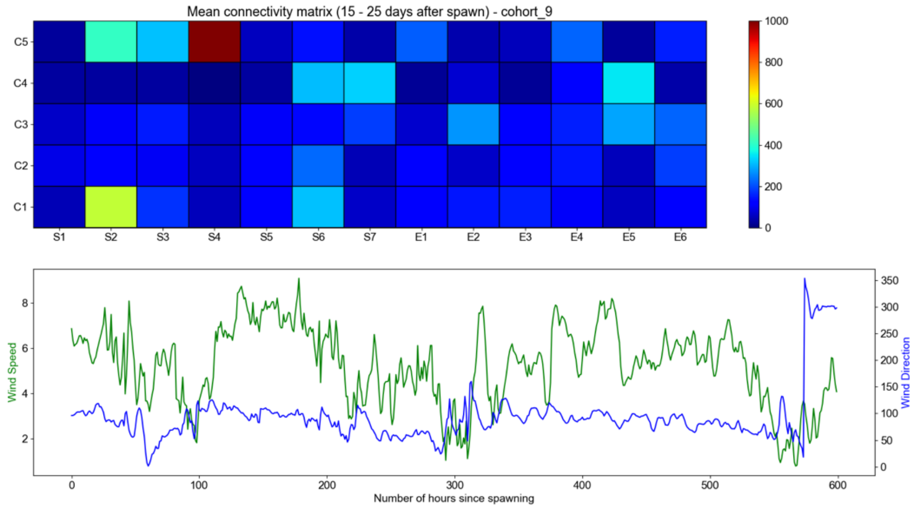
Mean connectivity matrix (15 - 25 days after spawn) - cohort_9
C5
C4
C3
C2
C1
S1 S2 S3 S4 S5 S6 S7 E1 E2 E3 E4 E5 E6
1000 800 600 400 200 0
Wind Speed
Wind Direction
Number of hours since spawning

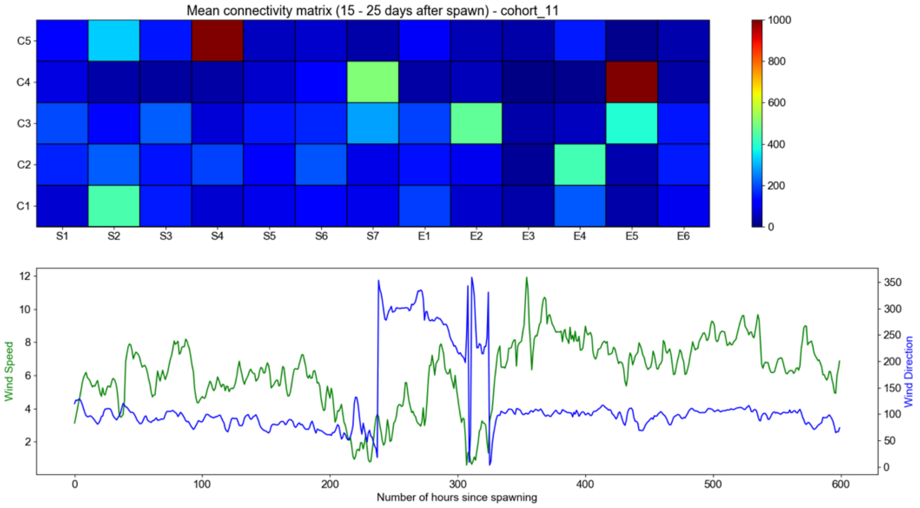
Mean connectivity matrix (15 - 25 days after spawn) - cohort_11
C5
C4
C3
C2
C1
S1 S2 S3 S4 S5 S6 S7 E1 E2 E3 E4 E5 E6
0 200 400 600 800 1000
Wind Speed
Wind Direction
Number of hours since spawning

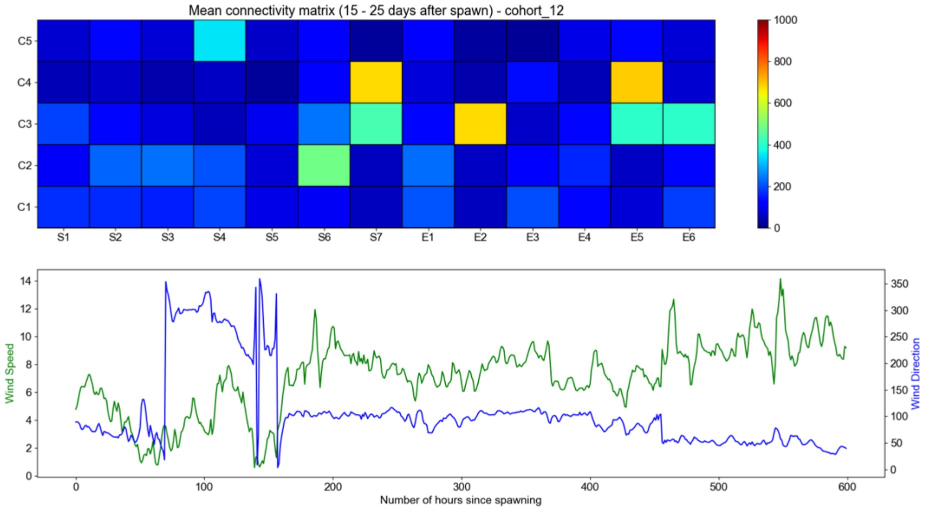
Mean connectivity matrix (15 - 25 days after spawn) - cohort_12
C5
C4
C3
C2
C1
S1 S2 S3 S4 S5 S6 S7 E1 E2 E3 E4 E5 E6
0 200 400 600 800 1000
Wind Speed
Wind Direction
Number of hours since spawning

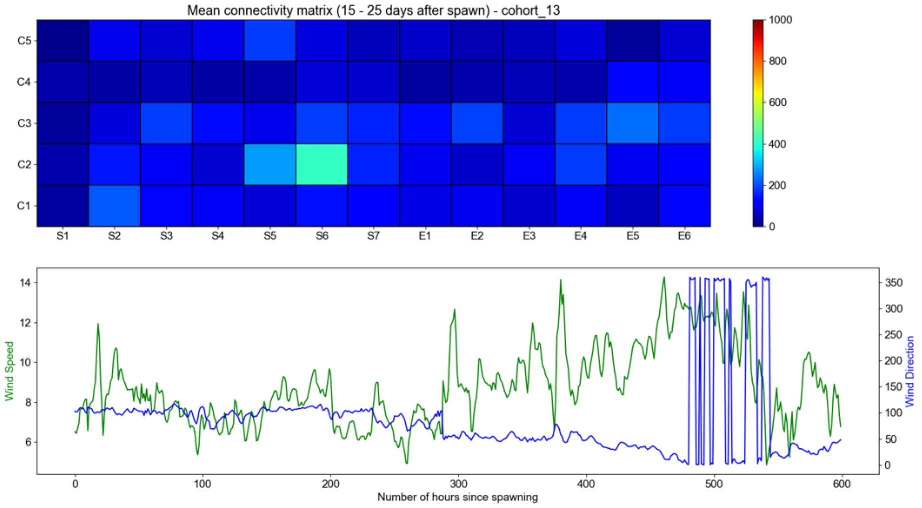
Mean connectivity matrix (15 - 25 days after spawn) - cohort_13
C5
C4
C3
C2
C1
S1 S2 S3 S4 S5 S6 S7 E1 E2 E3 E4 E5 E6
0 200 400 600 800 1000
Wind Speed
Wind Direction
Number of hours since spawning

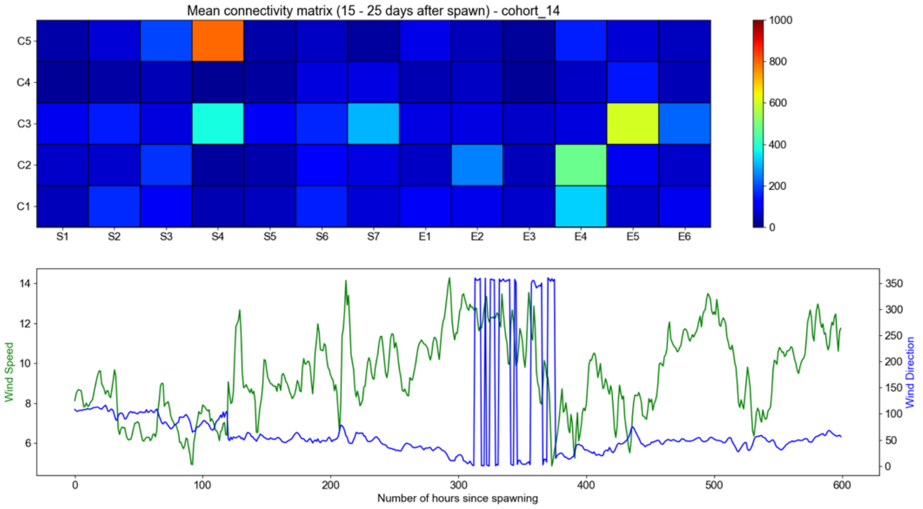
Mean connectivity matrix (15 - 25 days after spawn) - cohort_14
C5
C4
C3
C2
C1
S1 S2 S3 S4 S5 S6 S7 E1 E2 E3 E4 E5 E6
1000
800
600
400
200
0
Wind Speed
Wind Direction
Number of hours since spawning

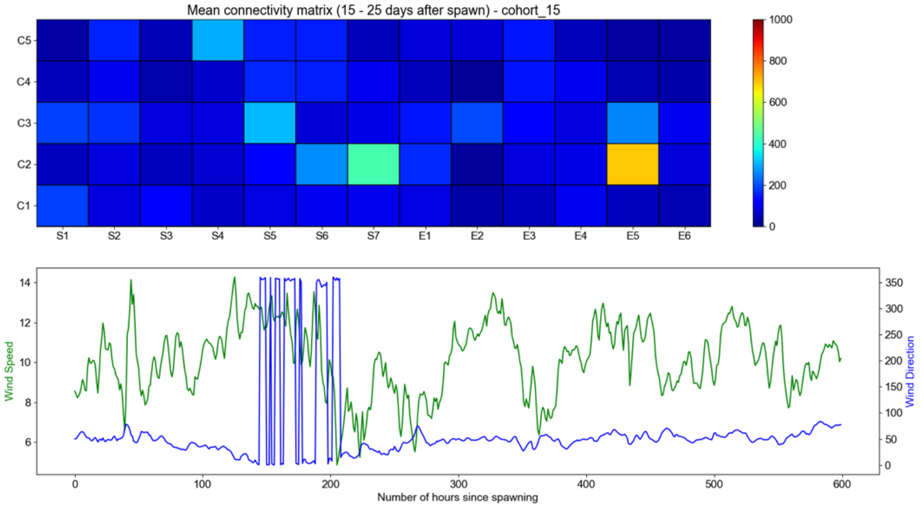
Mean connectivity matrix (15 - 25 days after spawn) - cohort_15
C5
C4
C3
C2
C1
S1 S2 S3 S4 S5 S6 S7 E1 E2 E3 E4 E5 E6
1000
800
600
400
200
0
Wind Speed
Wind Direction
Number of hours since spawning

Supplementary table S1: Annotation of the eight SNPs putatively selected.

Scaffold	position	prob	qval	alpha	fst	index	SNPeff	Transcripts impacted	Annotation	GO
**scaffold1138|size309401**	**130750**	0.97099	0.0043	2.4086	0.054	930	missense_variant; upstream_gene_variant	TRINITY_DN86686_c1_g1_i2; TRINITY_DN34861_c0_g2_i1	solute carrier organic anion transporter family member 4A1	GO:0005887; GO:0015347; GO:0015349; GO:0043252; GO:0042403
**scaffold1188|size238585**	**30706**	0.9986	0.0002	2.4297	0.052	1166	intergenic_region			
**scaffold1960|size257008**	**111339**	1	0	2.3826	0.049	3816	missense_variant	TRINITY_DN81573_c0_g1_i2	sushi domain-containing protein 2 isoform X1	GO:0070062; GO:0016021; GO:0005886; GO:0030247; GO:0005044; GO:0006955; GO:1902807; GO:0051782
**scaffold2247|size147876**	**82155**	1	0	2.869	0.074	4379	intergenic_region			
**scaffold2247|size147876**	**82182**	1	0	2.7206	0.066	4380	intergenic_region			
**scaffold3865|size74240**	**21548**	1	0	3.2283	0.098	7244	5_prime_UTR_variant	TRINITY_DN76995_c0_g1_i6	uncharacterized protein LOC105318238; contains a Caspase activation and recruitment domain: a protein-protein interaction domain	
**scaffold5372|size124137**	**33585**	1	0	2.2809	0.045	9230	downstream_gene_variant	TRINITY_DN26546_c0_g1_i1	pfu_aug1.0_172490.1_71976.t1 uncharacterized protein	
**scaffold6833|size89161**	**37067**	0.93019	0.0125	2.3482	0.053	10797	intergenic_region			

Supplementary Table S2 : Dun's Post Hoc results

Comparison	Z	P.unadj	P.adj
**Spats-Exploited**	-9.269	1.88E-20	5.63E-20
**Spats -Natural**	-6.171	6.81E-10	2.04E-09
**Exploited-Natural**	3.563	3.67E-04	1.10E-03
**Spats -Exploited**	-9.269	1.88E-20	1.13E-19
**Spats -Natural_NE**	-1.088	2.77E-01	1.00E+00
**Exploited-Natural_NE**	7.724	1.12E-14	6.74E-14
**Spats -Natural_SW**	-8.849	8.86E-19	5.32E-18
**Exploited-Natural_SW**	-1.314	1.89E-01	1.00E+00
**Natural_NE-Natural_SW**	-7.567	3.81E-14	2.29E-13
